# Substituting animal-based with plant-based foods—current evidence and challenges ahead

**DOI:** 10.1186/s44263-023-00036-z

**Published:** 2024-01-04

**Authors:** Daniel B. Ibsen

**Affiliations:** 1 Department of Public Health, Aarhus University, Aarhus, Denmark; 2 Steno Diabetes Center Aarhus, Aarhus University Hospital, Aarhus, Denmark; 3 Department of Nutrition, Sports and Exercise, University of Copenhagen, Frederiksberg, Denmark; 4 MRC Epidemiology Unit, University of Cambridge School of Clinical Medicine, Cambridge, UK

Recent findings from a systematic review and meta-analysis of cohort studies highlight the benefit of substituting some, but not all, animal-based foods with plant-based foods, providing crucial insights for policymakers amid the current climate and health crisis. I discuss the findings, research gaps and the need for standardised reporting going forward.

## Background

With the current global climate and health crisis, reducing human environmental impact and improving lifestyle habits are key priorities. Our dietary habits represent an important contributing factor. In fact, current food production systems contribute to about 21–37% of global greenhouse gas emissions [1], and unhealthy diets contribute substantially towards the rise in type 2 diabetes (T2D), cardiovascular disease (CVD) and mortality [2]. Animal-based foods in particular are reported to have a greater environmental impact [3], and some (e.g. processed meat), but not all (e.g. yogurt), have been linked with higher disease risk [4] compared with plant-based foods.

When changing diets, the concept of food substitutions becomes central. Imagine an intervention study that investigates the effect of replacing red meat with legumes. In practice, participants consuming red meat are advised to replace their intake of red meat with legumes. The comparison group may be advised to maintain their diet including the usual intake of red meat. Though intervention studies provide strong evidence, they are often limited to intermediate outcomes such as body weight, blood glucose, blood pressure and blood lipids. Cohort studies offer an advantage with longer follow-ups and incident disease outcomes. In contrast to the intervention studies, however, food substitution analyses in cohort studies utilise statistical modelling, most often comparing participants with different levels of intake across food types [5]. In recent years, methodological developments [5, 6] such as understanding the consequences of different adjustment strategies and more frequent application of food substitution methods in cohort studies have contributed to a growing evidence-based of such studies investigating animal- and plant-based foods.

Evidence synthesis in the form of systematic reviews and, if appropriate data from primary studies are available, meta-analysis forms the basis of current dietary recommendations such as the Nordic Nutrition Recommendations [7]. Previously, food-based dietary recommendations have been based on the synthesis of single food studies. With food substitutions being increasingly investigated, evidence synthesis is now possible. Such a synthesis was conducted by Neuenschwander et al. [8] investigating the replacement of animal- with plant-based foods and the risk of T2D, CVD and mortality. Given that dietary change involves substitution, and that the health impact of this substitution not only depends on what you consume but also what it replaces, this systematic review provides new insights but also highlights the gaps in the literature and showcases important methodological challenges.

## Replacing animal- with plant-based foods

The systematic review included 37 publications investigating the replacement of animal-based foods (red meat, processed meat, poultry, fish, eggs, dairy and butter) with plant-based foods (whole grains, nuts, legumes, vegetables, olive oil and margarine) [8]. All cohort studies in the meta-analysis used a single assessment of habitual dietary intake. Consequently, all substitutions were modelled based on the differences in the level of food intake between participants, not individual dietary change. Overall, the analysis found a lower risk of CVD when replacing processed meat with nuts, legumes or whole grains and when replacing eggs with nuts and butter with olive oil. The risk of T2D was lowered when replacing red meat with whole grains and nuts, replacing processed meat with nuts, poultry with whole grains and eggs with nuts or whole grains. Lastly, evidence of lower mortality rates was found when replacing red meat with nuts or legumes, replacing processed meat with nuts or legumes, dairy with nuts and eggs with nuts or legumes.

In summary, the consumption of nuts instead of most animal-based foods, apart from fish and dairy, and the intake of legumes or whole grains instead of red meat or processed meat were associated with improved long-term health. This aligns well with current dietary guidelines [7]. Nevertheless, what the findings also highlight is where there are gaps.

## Filling the gaps in evidence and methods

In fact, many substitutions were not investigated. For instance, no identified study investigated the replacement of dairy with plant-based foods except nuts. Whilst most foods classified as “healthy” plant-based foods (e.g. nuts, legumes, whole grains) were investigated, few studies investigated replacements with “unhealthy” plant-based foods (e.g. refined grains, French fries, sugar-sweetened beverages). Given that foods such as dairy and fish contribute important nutrients, yet also have a higher environmental impact than many plant-based foods, it is central to determine important trade-offs balancing health and environment. In addition, dairy includes a wide range of subtypes being either high-fat, low-fat, fermented or non-fermented. Each of these subtypes shows different health effects [9], arguing for more specific food group definitions.

Synthesising estimates from food substitution studies is complex as the interpretation of the individual estimates highly depends on the variables included in the statistical models of each study. All included substitutions in the meta-analysis were in grams consumed per day with adjustment of total energy intake in kilocalories per day [8]. This means that if there is a difference in the energy content between the substituted foods, the remaining energy must be balanced by the intake of other foods [5]. For instance, in order to replace 50 g/day of processed meat with 50 g/day of legumes, each contributing with different amounts of energy, the difference in energy must be compensated by other foods in the diet. Most of the combined studies adjusted for different sets of other foods. In addition, only the serving size of the food to be substituted was standardised to be the same across the studies. Therefore, the meta-analysed results must be interpreted carefully. For instance, a lower risk of CVD was observed for the replacement of 50 g/day of processed meat with different amounts of legumes and varying amounts of energy from other foods. Though not perfect, the approach of the meta-analysis by Neuenschwander et al. [8] is a step in the right direction. It would be possible to combine results from individual food substitution studies and avoid these pitfalls, but it relies on the reporting of the individual studies. One approach would be that each individual study modelled the food substitutions by including all individual food sources of total energy in a so-called all-components model [6]. Then, the variation from all other foods would be accounted for. If the individual estimates for each of the two foods being substituted and their variances and covariance were reported, then meta-analyses could standardise both foods to a specific energy intake across studies. This showcases the need for reporting guidelines of individual food substitution studies, as standardised reporting will improve future evidence synthesis (Table 1).

**Table 1:** Suggested additional reporting guidelines for individual food substitution studies and meta-analyses of food substitution studies

**Individual food substitution studies:**
• Aim: define your target food substitution, preferably described as a target intervention • Methods: describe and provide a rationale for the adjustment strategy in accordance with your target food substitution and explain the interpretation of different models, for instance, if the models are with and without adjustment for other foods not part of the substitutions explored • Results: report results from food substitution models using an all-components model, including their individual estimates, their variances and their co-variances to enable later meta-analyses
**Meta-analysis of food substitution studies:**
• Aim: define your target food substitution. Preferably described as a target intervention. • Methods: include considerations for how to handle different adjustment strategies across studies, if relevant, include subgroup meta-analyses of food substitution studies with different interpretations or sensitivity analyses that exclude certain studies • Discussion: discuss the interpretation of the meta-analysed estimate of the target food substitution, preferably in relation to the target intervention, and take the interpretation of the meta-analysed estimate into account when formulating the conclusion

## Conclusions

The health effect of what we eat not only depends on the food we eat but also what it replaces. The evidence from Neuenschwander et al. [8] suggests that the replacement of some animal-based foods such as red meat or processed meat with other plant-based foods such as nuts, legumes or whole grains shows a favourable effect on long-term health. However, there is still more to be investigated—particularly understanding the role of animal-based foods such as dairy and fish and the role of more “unhealthy” plant-based foods. As one of the first meta-analyses of food substitution studies, it is also evident that better reporting of individual food substitution studies is needed to improve future evidence syntheses.

## Data Availability

Not applicable.

## Abbreviations

CVD: Cardiovascular disease
T2D: Type 2 diabetes
